# The Impact of Reducing the Number of Wearable Devices on Measuring Gait in Parkinson Disease: Noninterventional Exploratory Study

**DOI:** 10.2196/17986

**Published:** 2020-10-21

**Authors:** Matthew Czech, Charmaine Demanuele, Michael Kelley Erb, Vesper Ramos, Hao Zhang, Bryan Ho, Shyamal Patel

**Affiliations:** 1 Digital Medicine & Translational Imaging Early Clinical Development Pfizer Inc Cambridge, MA United States; 2 Tufts Medical Center Boston, MA United States

**Keywords:** gait, Parkinson disease, wearable sensors, digital medicine

## Abstract

**Background:**

Measuring free-living gait using wearable devices may offer higher granularity and temporal resolution than the current clinical assessments for patients with Parkinson disease (PD). However, increasing the number of devices worn on the body adds to the patient burden and impacts the compliance.

**Objective:**

This study aimed to investigate the impact of reducing the number of wearable devices on the ability to assess gait impairments in patients with PD.

**Methods:**

A total of 35 volunteers with PD and 60 healthy volunteers performed a gait task during 2 clinic visits. Participants with PD were assessed in the On and Off medication state using the Movement Disorder Society version of the Unified Parkinson Disease Rating Scale (MDS-UPDRS). Gait features derived from a single lumbar-mounted accelerometer were compared with those derived using 3 and 6 wearable devices for both participants with PD and healthy participants.

**Results:**

A comparable performance was observed for predicting the MDS-UPDRS gait score using longitudinal mixed-effects model fit with gait features derived from a single (root mean square error [RMSE]=0.64; R2=0.53), 3 (RMSE=0.64; R2=0.54), and 6 devices (RMSE=0.54; R2=0.65). In addition, MDS-UPDRS gait scores predicted using all 3 models differed significantly between On and Off motor states (single device, *P*=.004; 3 devices, *P*=.004; 6 devices, *P*=.045).

**Conclusions:**

We observed a marginal benefit in using multiple devices for assessing gait impairments in patients with PD when compared with gait features derived using a single lumbar-mounted accelerometer. The wearability burden associated with the use of multiple devices may offset gains in accuracy for monitoring gait under free-living conditions.

## Introduction

Gait is a complex sensorimotor activity involving dynamic spatial-temporal coordination of legs, trunk, and arms. Gait impairments negatively impact the functional mobility of patients with Parkinson disease (PD) [1,2]. In the early stages of PD, gait impairments manifest as reduced gait speed, shorter stride lengths, gait asymmetry with higher variability of gait measures, and reduced amplitude of arm swing. As the disease progresses, gait measures become less asymmetric, but impairments continue to increase in severity. Worsening gait impairments coupled with balance and postural control issues lead to a significant reduction in mobility and an increased risk for falls in advanced PD [1,3,4].

Clinical assessment of gait in PD is limited to observational scales, such as the Movement Disorder Society version of the Unified Parkinson Disease Rating Scale (MDS-UPDRS), [5] and performance-based tests, such as the Timed Up and Go test [6]. While these tools have been clinically validated, assessments are influenced by the observer effect (Hawthorne effect) and quality of instructions [7]. Assessments are susceptible to rater bias; and because symptoms are rated on an ordinal scale, they lack the resolution to detect changes that occur on a continuum. In addition, since trained raters can only perform these assessments infrequently, they provide intermittent snapshots, which are inadequate for fully characterizing the day-to-day variability of symptoms [8].

Advances in wearable technology allow for the development of systems for objective measurement of gait [9-12]. Many of these systems, such as APDM Mobility Lab (APDM Inc) [13], provide a broad range of measures, quantifying various spatial and temporal aspects of gait. However, they generally require multiple sensing devices, which makes continuous, long-term monitoring difficult outside the lab or clinic. Recent research efforts to develop methods employing a single waist-mounted inertial sensing device (accelerometer and gyroscope) demonstrate the feasibility of monitoring gait in patients with mobility deficits, including PD, Huntington disease, poststroke disability, and sarcopenia [14-18]. Studies show moderate-to-good agreement between the frequency domain features (eg, dominant frequency amplitude or dominant frequency width) extracted from the accelerometer time series and subscales of the MDS-UPDRS associated with gait and balance [19,20]. However, unlike gait features like stride length and gait speed, these signal features do not have direct clinical meaning and are therefore, difficult to use for clinical decision making. Temporal (eg, swing time) and spatial (eg, stride length) gait features derived from a single accelerometer on the lower back (L5 vertebrae) have demonstrated moderate-to-excellent agreement with an instrumented walkway for 8 out of 14 gait parameters in healthy older adults and patients with PD [14]. Furthermore, gait features derived under free-living conditions had greater discriminative power than that of the laboratory-based gait assessments for differentiating between healthy older adults and patients with PD [21]. Compared to bilaterally worn ankle-mounted devices, lumbar-mounted accelerometers were satisfactory for measuring temporal gait features in young healthy adults despite their less accuracy [22].

While it is feasible to monitor gait using a single lumbar-mounted wearable device, the relationship between the number of devices used for deriving temporal and spatial gait features and their ability to detect clinically meaningful changes is not well understood. Herein, we employ a method, which relies on a single lumbar-mounted accelerometer that presents a significantly lower usability burden and affords better wearability compared to methods that rely on 3 or 6 devices [14,23-25]. However, the tradeoffs of reducing the number of devices may include lower accuracy in the estimation of gait features, measuring fewer aspects of gait, and reduced sensitivity for detection of clinically meaningful differences. Therefore, in order to objectively evaluate this tradeoff, we assessed (1) the accuracy and reliability of gait features derived using a single lumbar-mounted accelerometer relative to a reference system (APDM Mobility Lab) [13] and (2) the impact of reducing the number of sensing devices on the criterion and discriminative validity of gait features in patients with PD.

## Methods

### Study Participants

We recruited 35 participants with mild-to-moderate PD (Hoehn and Yahr scale score ≤3; mean age 68.3 years, SD 8.0 years; males, n=23; and females, n=12) and 60 healthy participants (mean age 44.1 years, SD 10.7 years; males, n=27; and females, n=33). Participants with PD took regular dopaminergic medication (levodopa-equivalent daily dose, mean 165.5 mg, SD 81.3 mg). Participants with PD were recruited and tested at Tufts Medical Center, Boston, Massachusetts. All procedures were approved by The Tufts Health Sciences Institutional Review Board (#12371). The protocol for the healthy cohort was approved by the Schulman Institutional Review Board (#201500837) and conducted at Pfizer, Andover, Massachusetts.

A participant with PD who self-reported as “On with dyskinesia” was excluded from the analysis since dyskinesia might interfere with gait feature measurements. Additionally, 1 healthy volunteer was removed from the analysis due to technical errors with data capture. The clinical and demographic characteristics of the participants whose data were available for analysis are listed in Table 1.

**Table 1:** Clinical and demographic characteristics.

Characteristics		Healthy participants (n=59)	Participants with PD^a^ (n=34)
Males, n		27	23
Females, n		32	11
Age (years), mean (SD)		44.4 (10.5)	68.1 (8.1)
Height (m), mean (SD)		1.7 (0.1)	1.7 (0.1)
BMI (Kg/m^2^), mean (SD)		25.3 (4.8)	28.9 (7.1)
**Participants with the Hoehn and Yahr stage of PD, n**			
	Hoehn and Yahr stage 1	N/A^b^	2
	Hoehn and Yahr stage 2	N/A	26
	Hoehn and Yahr stage 3	N/A	6
Levodopa-equivalent daily dose (mg/day), mean (SD)		N/A	164.5 (81.1)
**MDS-UPDRS^c^ III gait score in On condition, mean (SD)**			1.0 (0.9)
	Hoehn and Yahr stage 1	N/A	0.0 (0.0)
	Hoehn and Yahr stage 2	N/A	0.8 (0.7)
	Hoehn and Yahr stage 3	N/A	2.0 (0.9)
**MDS-UPDRS III gait score in Off condition, mean (SD)**			1.4 (0.9)
	Hoehn and Yahr stage 1	N/A	0.0 (0.0)
	Hoehn and Yahr stage 2	N/A	1.2 (0.7)
	Hoehn and Yahr stage 3	N/A	2.7 (0.5)

^a^PD: Parkinson disease.

^b^N/A: Not applicable.

^C^MDS-UPDRS: Movement Disorder Society version of the Unified Parkinson Disease Rating Scale.

### Device Setup

As illustrated in Multimedia Appendix 1A, participants were instrumented with 6 wearable devices (Opal, APDM Inc) located bilaterally on the wrist and foot, and at the lumbar (approximately at the L5 vertebra) and sternum locations. Each device recorded raw data from 9-axis inertial sensors (triaxial accelerometer, triaxial gyroscope, and triaxial magnetometer) at a sampling rate of 128 Hz.

### Experimental Protocol

Participants performed a battery of physical activities and cognitive tasks over the course of 2 visits. Both visits were identical for healthy participants but were randomized for participants with PD so that they were in the On state (about 1 hour after medication intake, confirmed with the patient self-report and clinician report) during 1 visit and in the Off state (about 3 hours after last medication intake, confirmed with the patient self-report and clinician report) during the other visit. Physical activities during each visit included scripted activities of daily living (eg, tying a shoe, opening and closing a door) and motor assessments from the MDS-UPDRS part III (eg, 2-minute gait task, finger tapping). In this paper, we present the analysis based on the data collected during the 2-minute gait task. This is to ensure uniform testing conditions for determining the agreement of postexperiment sensor data processing. During this gait task, participants were instructed to walk back and forth along a straight 10-meter track at a comfortable pace for a period of 2 minutes. Participants with PD were assigned an MDS-UPDRS gait score on an ordinal scale of 0 to 4 by a neurologist to assess the degree of gait impairment. Sample sizes (n) for MDS-UPDRS gait scores of 0, 1, 2, and 3 across both visits were 17, 27, 18, and 6, respectively.

### Gait Feature Extraction

APDM Mobility Lab is a commercially available system widely used for objective assessment of gait and leverages data from 3 to 6 wireless, body-worn Opal inertial devices [13,26]. We used APDM Mobility Lab to derive a set of lower limbs, lumbar, and trunk range of motion and upper limb gait features from 6 wearable devices placed on the lower back, sternum, and bilaterally on the feet and wrists (Multimedia Appendix 2). Using 3 sensors located on the lower back and both feet, APDM Mobility Lab can only derive features related to the lower limb and lumbar range of motion. Therefore, we used only features related to lower limb and lumbar range of motion as the 3-sensor feature set (Multimedia Appendix 2). To derive gait features from a single lumbar-mounted triaxial accelerometer, we developed and implemented a previously published wavelet-based method [14] in a Python v3.6 package called GaitPy (Multimedia Appendix 1) [25]. A complete list of gait features derived from a single lumbar-mounted device and those requiring additional devices can be seen in Multimedia Appendix 2.

### Statistical Methods

Statistical analysis was performed in R, version 3.4.1 (The R Project for Statistical Computing) [27], using the following packages: “psych” for intraclass correlation coefficient (ICC), “BlandAltmanLeh” for Bland-Altman plots, “nlme” for linear mixed-effects model, “car” for type 3 analysis of variance, and “MASS” for stepwise model selection.

**Figure 1:**
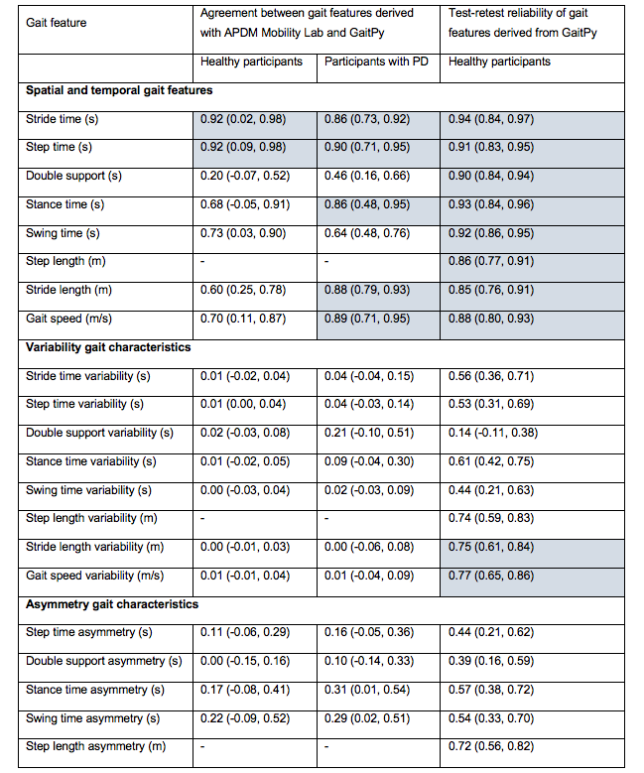
Agreement between gait features derived using the APDM Mobility Lab and GaitPy and test-retest reliability of gait features derived with GaitPy in healthy participants. Intraclass correlation coefficient values showing excellent agreement (between 0.75 and 1) are highlighted in blue. PD: Parkinson disease.

The median value of each gait feature extracted from the data collected during the 2-minute walking task was calculated for each visit separately. Test-retest reliability of gait features was assessed by calculating the ICC on data collected from healthy volunteers during visit 1 and visit 2. ICC was also used in addition to Bland-Altman plots and 95% limits of agreement to evaluate the agreement between gait features derived using GaitPy and APDM Mobility Lab. The results are presented in Figure 1, where values are ICC_2,1_ coefficient (2-way random effects, absolute agreement) with lower and upper confidence bounds, reported as ICC coefficient (lower, upper). Test-retest reliability and agreement between features were assessed according to the following benchmarks. ICC ≤ 0.4 indicates “poor,” 0.4 to 0.59 “moderate,” 0.6 to 0.74 “good,” and 0.75 to 1 “excellent” reliability [28]. Variation of gait features with the MDS-UPDRS gait score in patients with PD was assessed using the Kruskal-Wallis test. Posthoc Conover-Iman tests were used for pairwise comparisons, and multiplicity was adjusted using false discovery rate correction.

Gait features derived using a single, 3, and 6 devices were separately used to fit 3 longitudinal mixed-effects regression models to predict the clinician’s MDS-UPDRS gait score (using the *lme* function in “nlme” R package). Prior to model fitting, pairwise correlation between sensor features was computed, and highly correlated features were removed. Gait features and covariates including age, gender, visit number, BMI, and years since first symptoms were modeled as fixed effects and participant as a random effect. An unstructured correlation matrix was used. Numerical features were standardized to have 0 mean and unit variance. Stepwise model selection was performed using Akaike Information Criterion as a cost function to achieve the optimal model fit (using the *stepAIC* function in “MASS” R package). Analysis of variance findings were reported as chi-square values and corresponding *P* values using Type 3 sum of squares (statistics were derived using the “car” R package). Final models were used to predict the clinician’s score using leave-1-subject-out cross-validation. We report the root mean square error (RMSE) and marginal *R*^2^ representing the variance explained by the model of fixed effects. Paired Wilcoxon signed rank tests were used to compare predicted gait scores between On and Off states.

### Ethical Compliance

The study of participants with PD was approved by The Tufts Health Sciences Institutional Review Board (#12371) and conducted at the Tufts Medical Center. The study of healthy participants was approved by the Schulman Institutional Review Board (#201500837), and conducted at Pfizer in Andover, Massachusetts. Written informed consent was obtained from all participants prior to testing. We confirm that we have read the journal’s position on issues involved in ethical publication and affirm that this work is consistent with those guidelines.

## Results

### Accuracy of Gait Features Derived Using a Single Device

Gait features derived during the 2-minute walking task using GaitPy (single lumbar-mounted device) were compared with the same features derived using the APDM Mobility Lab (6 devices) for healthy participants and participants with PD with data from their 2 visits. Gait features derived using the APDM Mobility Lab were used as the reference since the APDM device has been validated against data from an instrumented treadmill and has been extensively used in both healthy populations and populations with PD [26,29]. Excellent agreement was observed between the 2 methods for stride time and step time in both healthy participants and participants with PD (ICC≥0.86), as shown in Figure 1. Furthermore, excellent agreement was observed for stance time, stride length, and gait speed in participants with PD (ICC=0.86, 0.88, and 0.89, respectively), and agreement was good in healthy participants (ICC=0.68, 0.60, and 0.70, respectively) (Figure 1). Bland-Altman analysis showed that mean difference between GaitPy and APDM Mobility Lab was smaller for longer stance times (Multimedia Appendix 3). Good agreement was also observed for swing time in both healthy participants and participants with PD (0.64≤ICC≤0.73) (Figure 1). In contrast, double support showed poor agreement in healthy participants (ICC=0.20) and moderate agreement in participants with PD (ICC=0.46) (Figure 1). Asymmetry and variability features also showed poor agreement (ICC≤0.31) between the 2 methods for both healthy participants and participants with PD (Figure 1).

### Reliability of Gait Features Derived Using a Single Device

Test-retest reliability of gait features derived using GaitPy was assessed using the data collected from healthy participants. Excellent test-retest reliability (ICC≥0.85) (Figure 1) was observed for all spatial and temporal gait features. Asymmetry and variability features showed poor-to-excellent test-retest reliability (0.14≤ICC≤0.77) (Figure 1).

### Criterion Validity of Sensor-Derived Gait Features

We assessed the ability of gait features derived by using methods relying on different device setups (single device, 3 devices, and 6 devices) to distinguish between MDS-UPDRS gait scores. Based on the APDM Mobility Lab documentation [30], we could determine which gait features are available for analysis with a 3-device setup and a 6-device setup. Therefore, for the purpose of this comparison, we limited our analysis to the 1-device setup (using GaitPy) and 3- and 6-device setups (using APDM Mobility Lab).

**Figure 2:**
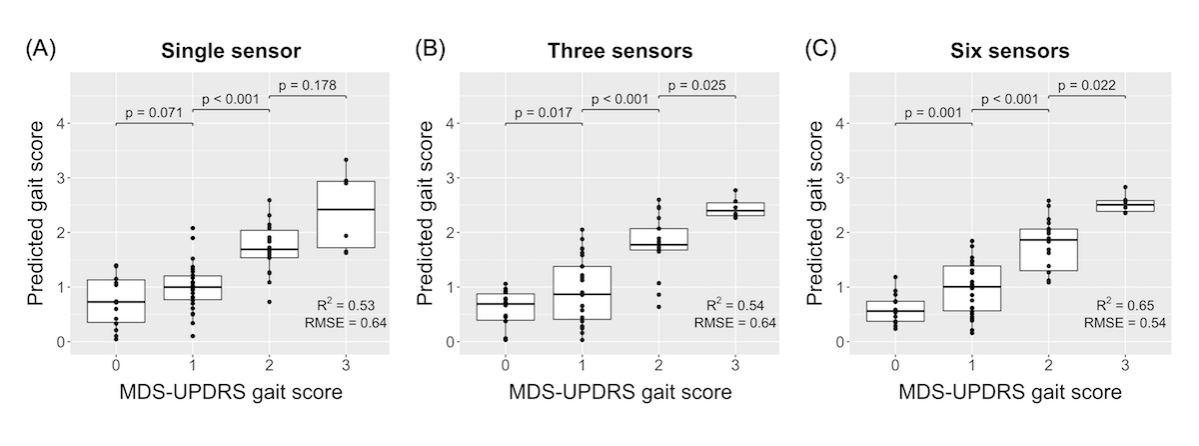
MDS-UPDRS gait score model performance fit using gait features from (A) single device at the lumbar (L5) location (GaitPy), (B) 3 devices (APDM Mobility Lab), and (C) 6 devices (APDM Mobility Lab). MDS-UPDRS: Movement Disorder Society version of the Unified Parkinson Disease Rating Scale.

The spatial features of gait (ie, gait speed, stride length, and step length) varied most significantly with MDS-UDPRS gait scores in participants with PD (Multimedia Appendix 4). Using leave-1-subject-out cross-validation, the longitudinal mixed-effects regression model based on gait features derived using a single lumbar-mounted device predicted the clinician’s gait score with an RMSE=0.64 and an *R*^2^=0.53. The predicted score significantly distinguished between scores of 1 and 2 (*P*<.001), and marginally distinguished between scores of 0 and 1 (*P*=.07), and 2 and 3 (*P*=.18) (Figure 2A). Stance time (*χ*^2^_1_=12.8; *P*<.001), step length (*χ*^2^_1_=49.2; *P*<.001), and step length asymmetry (*χ*^2^_1_=6.7; *P*=.01) had significant effects on describing the MDS-UPDRS gait score.

Comparable performance was observed for a model based on gait features derived using data from 3 devices (RMSE=0.64; *R*^2^=0.54). The *R*^2^ value for the 3-device model was only slightly higher than the single-device model. The predicted gait score could significantly distinguish between MDS-UPDRS gait scores of 0 and 1 (*P*=.02), 1 and 2 (*P*<.001), and 2 and 3 (*P*=.03) (Figure 2B). Pitch at initial contact (*χ*^2^_1_=7.3; *P*=.007), maximum pitch (*χ*^2^_1_=10.5; *P*=.001), cadence (*χ*^2^_1_=14.9; *P*<.001), initial mid-swing duration (*χ*^2^_1_=4.5; *P*=.03), and pitch at toe off variability (*χ*^2^_1_=6.4; *P*=.011) had a significant effect on describing the MDS-UPDRS gait score.

The model based on gait features derived using data from 6 devices achieved better performance than the single and 3-device model at predicting clinician gait score (RMSE=0.54; *R*^2^=0.65). The predicted gait score significantly distinguished between MDS-UPDRS gait scores of 0 and 1 (*P*=.001), 1 and 2 (*P*<.001), and 2 and 3 (*P*=.02) (Figure 2C). Pitch at initial contact (*χ*^2^_1_=8.9; *P*=.003), maximum pitch (*χ*^2^_1_=5.4; *P*=.02), cadence (*χ*^2^_1_=19.8; *P*<.001), initial mid-swing duration asymmetry (*χ*^2^_1_=5.7; *P*=.02), trunk sagittal average angle (*χ*^2^_1_=18.9; *P*<.001), upper limb foot phase difference (*χ*^2^_1_=5.0; *P*=.03), maximum pitch variability (*χ*^2^_1_=8.6; *P*=.003), trunk sagittal average angle variability (*χ*^2^_1_=5.0; *P*=.025), BMI (*χ*^2^_1_=10.0; *P*=.002), and years since first symptom (*χ*^2^_1_=7.0; *P*=.008) had significant effects on describing the MDS-UPDRS gait score.

### Discriminative Validity of Sensor-Derived Gait Features

**Figure 3:**
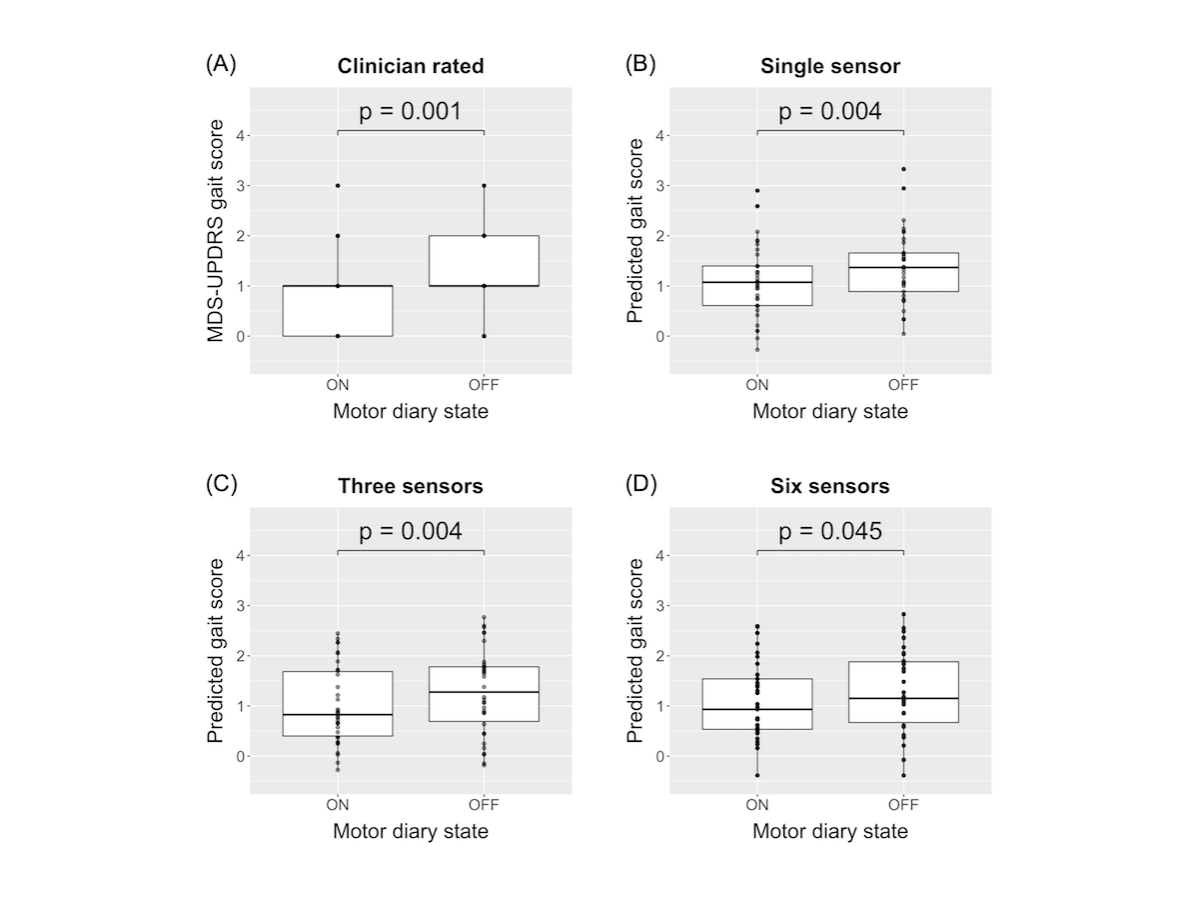
Distribution of the (A) clinician-rated gait score, (B) single device predicted gait score, (C) 3-device predicted gait score, and (D) 6-device predicted gait score, grouped by patient-reported On and Off motor states. MDS-UPDRS: Movement Disorder Society version of the Unified Parkinson Disease Rating Scale.

We assessed the ability of predicted gait scores derived using methods relying on different device setups (single device, 3 devices, and 6 devices) to discriminate between On and Off motor states. As shown in Figure 3A, the clinician-rated MDS-UPDRS gait score was significantly different (*P*=.001) between the patient-reported On and Off state. Similarly, predicted gait scores, estimated from 1-, 3-, and 6-device models (Figure 3B-D), all significantly differentiated between On and Off states (*P*=.004, *P*=.004, and *P*=.045 respectively).

## Discussion

### Principle Findings

In this exploratory, noninterventional study involving healthy participants and participants with PD, we derived gait features from participants during 2 clinic visits using wearable devices. We found that gait features derived from a single lumbar-mounted accelerometer could predict the clinician-rated gait impairment score to a similar degree as gait features derived from 3 or 6 sensors. Additionally, analogous to clinician-rated scores, predicted gait scores using gait features derived from either 1, 3, or 6 devices all significantly distinguished between the On and Off medication states. Our results suggest that a subset of gait features, derivable using a single lumbar-mounted accelerometer, may be sufficient to measure the degree of gait impairment and the effects of treatment in patients with PD.

### Accuracy and Reliability of Gait Features Derived Using a Single Device

Agreement of GaitPy with the reference system (APDM Mobility Lab) was assessed in both healthy participants and participants with PD. While we observed moderate-to-excellent agreement between the most temporal and spatial features of gait derived using GaitPy from a single device and gait features provided by APDM Mobility Lab using 6 devices, the agreement was poor for asymmetry and variability features. Notably, agreement was better in participants with PD for 4 out of 7 temporal and spatial features. The differences between ICC values for healthy participants and participants with PD were significant for stance time (0.86 vs 0.68), stride length (0.88 vs 0.60), and gait speed (0.89 vs 0.70). This result contrasts a prior study [14] where a good agreement with the reference system (gait mat) for both participants with PD and age-matched healthy controls was observed. However, unlike the prior study [14], the 2 groups in our study were not age-matched. To this end, patients with PD showed a wider range of values for gait speed and stride length compared to healthy participants, especially in the lower range, which may have contributed to a better agreement (Multimedia Appendix 5B and C).

We evaluated the test-retest reliability of gait parameters derived using GaitPy in a sample of 59 healthy participants. Test-retest reliability for GaitPy was excellent for all spatial and temporal features, whereas it was poor-to-excellent for asymmetry and variability features. These results suggest that the temporal and spatial features of gait can be reliably measured using a single accelerometer mounted on the lower back. However, as has been reported previously [14], the agreement and reliability of variability and asymmetry features might be sensitive to the employed measurement technique (eg, sensing modality or device location). This is partially because asymmetry and variability are small measurements, which are significantly affected by noise or error in the measurement of temporal or spatial features. Potential sources of measurement error for GaitPy include (1) biomechanical approximation of the inverted pendulum model, (2) error in the estimation of vertical displacement from vertical acceleration, and (3) distal location of the sensing device relative to the feet.

### Tradeoffs Between Gait Features Derived Using Different Device Setups

We assessed the criterion and discriminative validity of MDS-UPDRS gait scores using linear mixed-effects models based on gait features derived using data from a single device, 3 devices, and 6 devices. Although a single device provides substantially fewer features of gait compared to either 3- or 6-device models, 17 of the 34 features that varied most significantly (*P*=.004) with MDS-UPDRS gait score can be derived using a lumbar-mounted sensor (Multimedia Appendix 4). This includes many gait features known to be affected in PD, including stance time, gait speed, step-to-step asymmetries, and gait variability [3,4].

While the 6-device model (RMSE=0.54; *R*^2^=0.65) performed slightly better at estimating MDS-UPDRS gait score, performance of the 3-device model (RMSE=0.64; *R*^2^=0.54) was comparable to the single device model (RMSE=0.64; *R*^2^=0.53). However, unlike the 3 and 6 device models, the single device model was unable to significantly distinguish between the adjacent scores such as 0 and 1 or 2 and 3. A potential reason for this could be the small number of observations for class 3 (n=6). Additionally, gait features related to the pitch and mid-swing duration that were significant for both the 3 and 6-device models could not be derived using the single device model. This indicates features derived from the lower extremity (eg, foot) might have a higher predictive power. Indeed, 3 of 10 features in the 6-device model and 3 of 5 features in the 3-device model that were significant were related to the pitch of foot.

When we assessed the ability of gait scores predicted by the linear mixed-effects models to differentiate between On and Off motor states, we found significant differences (*P*=.045) for gait scores derived using 1-, 3-, and 6-device models. Additionally, the Off to On gait score directionality was largely consistent between those produced by each model and the clinician-rated gait score. In 10 of the 12 subjects, the clinician score and the predicted score differences between On and Off states were in the same direction (Multimedia Appendix 5A). This was comparable with the 3-device model (10/12) (Multimedia Appendix 5B) and 6-device model (10/12) (Multimedia Appendix 5C).

### Limitations

Data analyzed in this study were collected during performance of motor assessments in the laboratory settings and could be affected by the observer effect and heightened awareness of the patient. Gait features derived using wearable devices were not validated against a gold-standard reference (eg, an instrumented walkway or a motion capture system). This limitation of our work is mitigated to some extent by prior work in which the authors evaluated the algorithm implemented in GaitPy against an instrumented walkway [31]. Another limitation of our work is that the healthy participants and participants with PD were not age matched. Therefore, the results for accuracy and reliability in our healthy cohort might be different in healthy older adults. Additional work is required to validate the results presented herein on an independent data set as well as to confirm the ability of GaitPy to accurately assess gait impairment in free-living conditions.

### Conclusion

Our results suggest that a single triaxial accelerometer on the lower back may be sufficient to characterize gait impairments in patients with PD. Algorithms that estimate gait features from a lumbar-mounted sensor, such as GaitPy, could provide clinically meaningful measures of changes in the severity of gait impairments and changes in motor state associated with the effects of treatment in patients with PD. The long battery life of an accelerometer-only device and high degree of utility associated with a single device worn on the lower back enable further investigations to assess the validity of this approach for monitoring gait under free-living conditions. Comparing sensor-derived gait features with classical patient-reported motor diary–based approaches in their ability to detect treatment-related effects may provide an insight into the utility of a single lumbar-mounted sensor in free-living environments. Our ongoing efforts are focused on performing a clinical validation in a semisupervised setting as an intermediate step between the clinic and at-home environment.

## Appendix

Multimedia Appendix 1 Participants instrumented with six wearable devices (Opal, APDM, Inc) located bilaterally on the wrist and foot, and at the lumbar (approximately at the L5 vertebra) and sternum. *Adapted with permission from APDM Wearable Technologies.

Multimedia Appendix 2 Gait features derived using GaitPy with a single lumbar-mounted device, APDM Mobility Lab with 3 devices, and APDM Mobility Lab with 6 devices. *Step length is not calculated by APDM Mobility Lab.

Multimedia Appendix 3 Bland-Altman analysis comparing (A) stance time, (B) stride length. and (C) gait speed agreement between GaitPy and APDM Mobility Lab in healthy volunteers (HV) and patients with Parkinson disease (PD).

Multimedia Appendix 4 Kruskal-Wallis rank sum statistics and *P* values for sensor-derived features of gait in participants with Parkinson disease that varied significantly (P≤.01) with MDS-UPDRS gait score in order of significance.

Multimedia Appendix 5 A comparison between MDS-UPDRS gait scores and predicted score changes between (Off - ON) visits in patients with Parkinson disease.

## Abbreviations

ICC: intraclass correlation coefficient
MDS-UPDRS: Movement Disorder Society version of the Unified Parkinson Disease Rating Scale
PD: Parkinson disease
RMSE: root mean square error
