# Supplementary figures and images for: The Impact of Reducing the Number of Wearable Devices on Measuring Gait in Parkinson Disease: Noninterventional Exploratory Study
Source: JMIR Rehabil Assist Technol. 2020 Oct 21;7(2):e17986. doi: 10.2196/17986 (PMC7641789; doi:10.2196/17986)

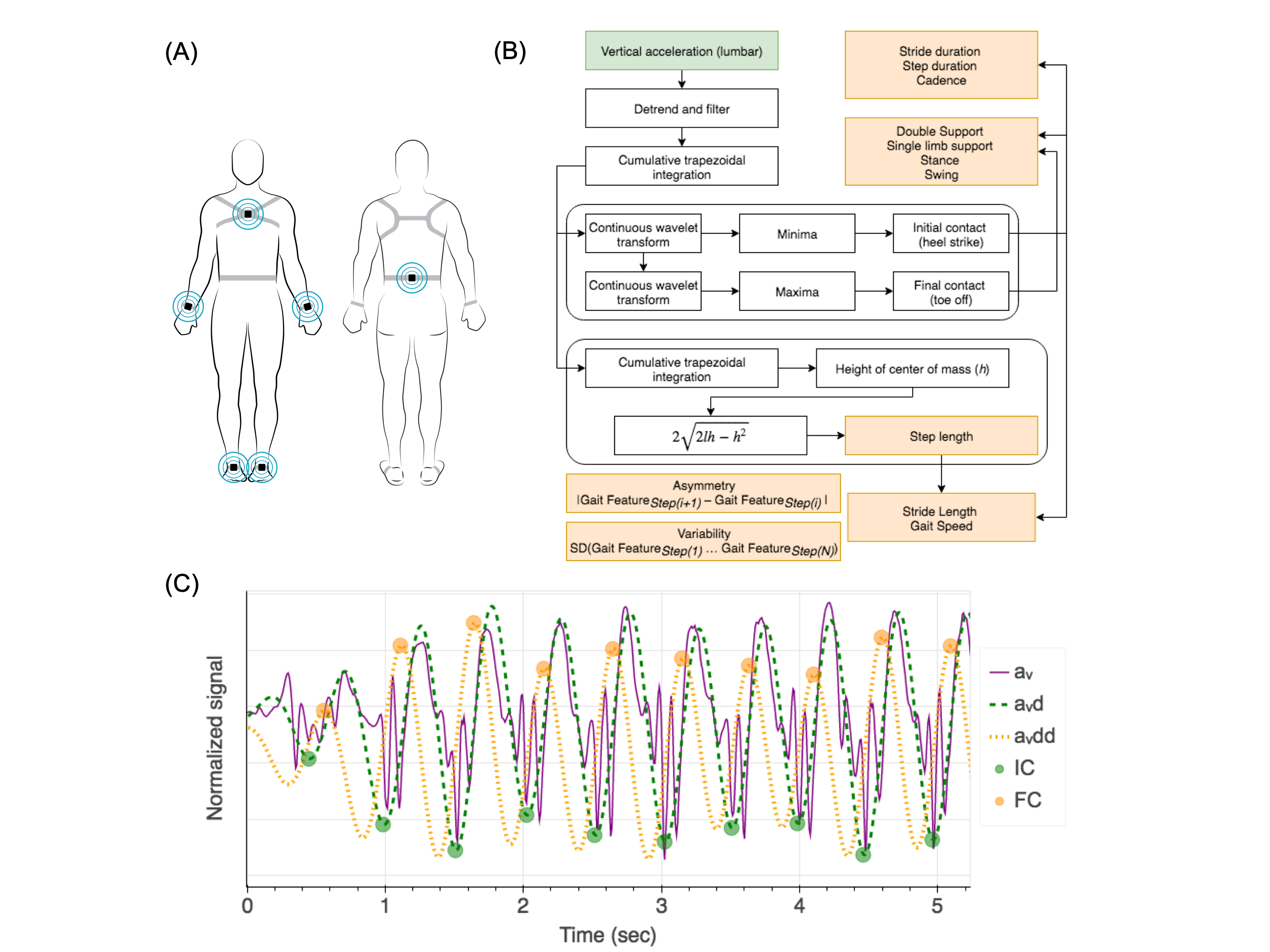

Supplement: Multimedia Appendix 1 [file rehab_v7i2e17986_app1.png]

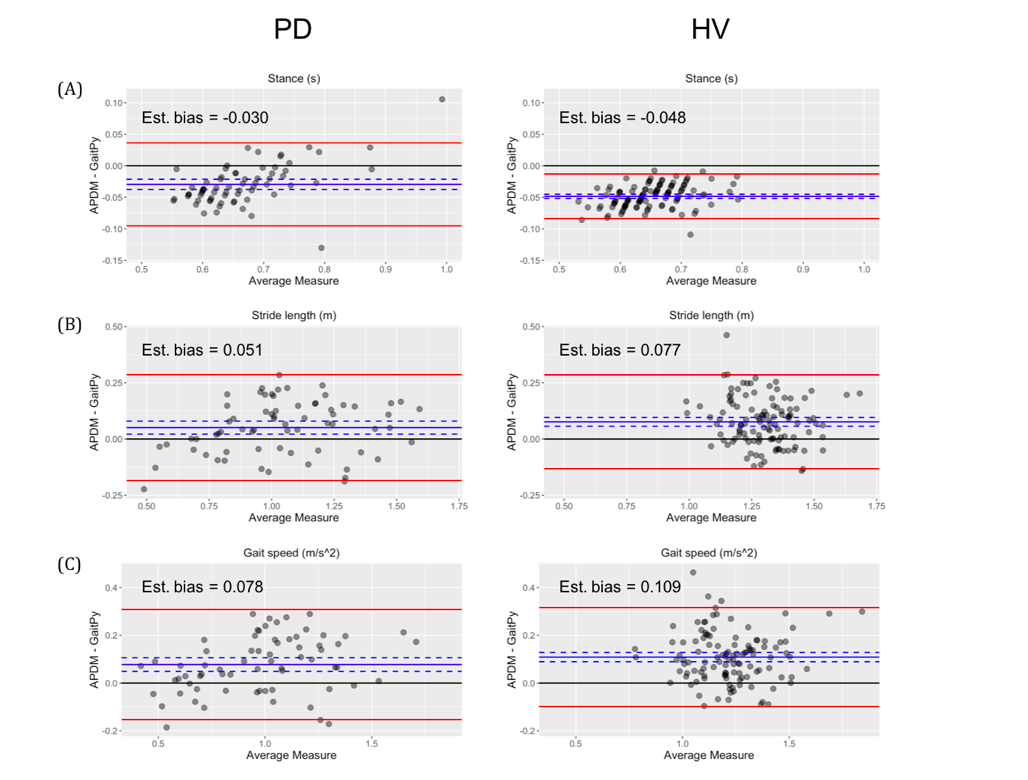

Supplement: Multimedia Appendix 3 [file rehab_v7i2e17986_app3.png]

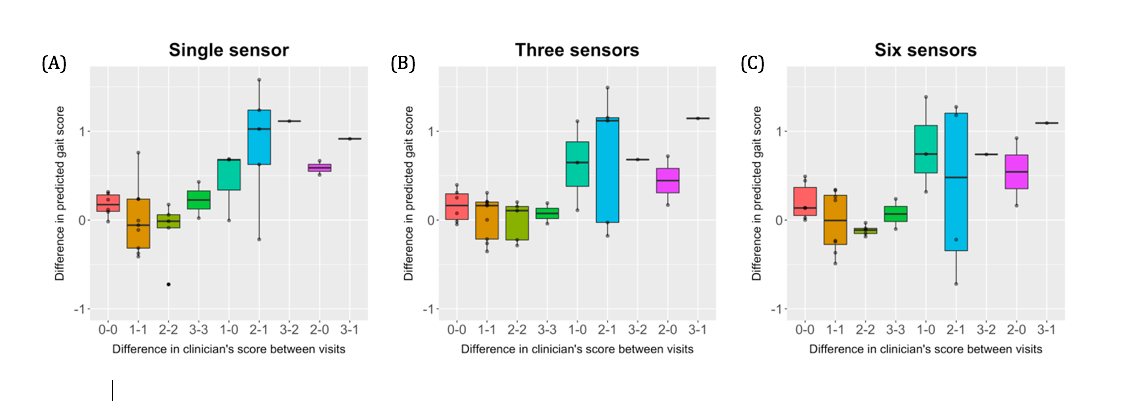

Supplement: Multimedia Appendix 5 [file rehab_v7i2e17986_app5.png]
