# Supplementary material for: The Impact of Reducing the Number of Wearable Devices on Measuring Gait in Parkinson Disease: Noninterventional Exploratory Study
Source: JMIR Rehabil Assist Technol. 2020 Oct 21;7(2):e17986. doi: 10.2196/17986 (PMC7641789; doi:10.2196/17986)
Supplement: Multimedia Appendix 2 [file rehab_v7i2e17986_app2.docx]

| **Gait features** | **1 Device (lumbar)** | **3 Devices (lumbar and both feet)** | **6 Devices (lumbar, both feet, both wrists, and sternum)** |
| --- | --- | --- | --- |
| **Lower Limb Features** | | | |
| Stride Time (s) | ✔ | ✔ | ✔ |
| Cadence (steps/min) | ✔ | ✔ | ✔ |
| Step Time (s) | ✔ | ✔ | ✔ |
| Stance Time (s) | ✔ | ✔ | ✔ |
| Swing Time (s) | ✔ | ✔ | ✔ |
| Initial Double Support (s) | ✔ | ✔ | ✔ |
| Terminal Double Support (s) | ✔ | ✔ | ✔ |
| Double Support (s) | ✔ | ✔ | ✔ |
| Single Limb Support (s) | ✔ | ✔ | ✔ |
| Step Length (m) | ✔ | * | * |
| Stride Length (m) | ✔ | ✔ | ✔ |
| Gait Speed (m/s) | ✔ | ✔ | ✔ |
| Elevation at Mid Swing (cm) |  | ✔ | ✔ |
| Lateral Step Deviation (cm) |  | ✔ | ✔ |
| Lateral Swing Max (cm) |  | ✔ | ✔ |
| Initial + Mid Swing Time (s) |  | ✔ | ✔ |
| Maximum Pitch (degrees) |  | ✔ | ✔ |
| Pitch at Initial Contact (degrees) |  | ✔ | ✔ |
| Pitch at Mid Swing (degrees) |  | ✔ | ✔ |
| Pitch at Tow Off (degrees) |  | ✔ | ✔ |
| Terminal Swing Time (s) |  | ✔ | ✔ |
| Toe Out Angle (degrees) |  | ✔ | ✔ |
| Toe Out Angle Max (degrees) |  | ✔ | ✔ |
| Toe Out Angle Min (degrees) |  | ✔ | ✔ |
| **Lumbar Range of Motion** | | | |
| Coronal Range of Motion (degrees) |  | ✔ | ✔ |
| Sagittal Range of Motion (degrees) |  | ✔ | ✔ |
| Transverse Range of Motion (degrees) |  | ✔ | ✔ |
| **Trunk Range of Motion** | | | |
| Coronal Range of Motion (degrees) |  |  | ✔ |
| Sagittal Range of Motion (degrees) |  |  | ✔ |
| Transverse Range of Motion (degrees) |  |  | ✔ |
| **Upper Limb Features** | | | |
| Foot Phase Difference (degrees) |  |  | ✔ |
| Maximum velocity (degrees/s) |  |  | ✔ |
| Range of motion (degrees) |  |  | ✔ |

**Multimedia Appendix 2.** Gait features derived using GaitPy with a single lumbar-mounted device, APDM Mobility Lab with 3 devices, and APDM Mobility Lab with 6 devices.
