# Supplementary material for: The Impact of Reducing the Number of Wearable Devices on Measuring Gait in Parkinson Disease: Noninterventional Exploratory Study
Source: JMIR Rehabil Assist Technol. 2020 Oct 21;7(2):e17986. doi: 10.2196/17986 (PMC7641789; doi:10.2196/17986)
Supplement: Multimedia Appendix 4 [file rehab_v7i2e17986_app4.docx]

**Multimedia Appendix 4.** Kruskal-Wallis rank sum statistics and P values for sensor-derived features of gait in participants with Parkinson disease that varied significantly (P≤.01) with MDS-UPDRS gait score in order of significance.

| Gait feature | PD | |
| --- | --- | --- |
|  | Kruskal-Wallis rank sum statistic | p-value |
| GaitPy (single device) | | |
| Gait speed | 41.66 | < 0.001 |
| Stride length | 34.94 | < 0.001 |
| Step length | 34.75 | < 0.001 |
| Swing time | 25.79 | < 0.001 |
| Stride time | 25.27 | < 0.001 |
| Gait speed var | 24.76 | < 0.001 |
| Double support | 24.32 | < 0.001 |
| Step time | 24.12 | < 0.001 |
| Stance time | 23.68 | < 0.001 |
| Stance asymmetry | 18.90 | < 0.001 |
| Swing asymmetry | 17.60 | < 0.001 |
| Step time asymmetry | 15.72 | 0.001 |
| Stance time var | 14.46 | 0.002 |
| APDM Mobility Lab (3 or 6 devices) | | |
| Gait speed | 34.98 | < 0.001 |
| Stride length | 33.22 | < 0.001 |
| Step time var | 32.77 | < 0.001 |
| Stride time var | 32.55 | < 0.001 |
| Pitch at initial contact | 26.84 | < 0.001 |
| Pitch at toe off | 25.76 | < 0.001 |
| Maximum pitch | 25.17 | < 0.001 |
| Double support var | 25.13 | < 0.001 |
| Initial mid swing var | 25.08 | < 0.001 |
| Trunk relative transverse range of motion | 24.51 | < 0.001 |
| Stance time var | 23.91 | < 0.001 |
| Swing time var | 23.91 | < 0.001 |
| Terminal swing var | 22.53 | < 0.001 |
| Single limb support var | 21.58 | < 0.001 |
| Step time | 21.02 | < 0.001 |
| Step time asymmetry | 20.71 | < 0.001 |
| Stride time | 20.38 | < 0.001 |
| Cadence | 20.38 | < 0.001 |
| Maximum velocity | 19.85 | < 0.001 |
| Terminal swing asymmetry | 18.55 | < 0.001 |
| Lumbar coronal range of motion | 17.07 | 0.001 |
| Initial double support var | 16.51 | 0.001 |
| Upper limb range of motion | 16.50 | 0.001 |
| Stance asymmetry | 15.70 | 0.001 |
| Swing asymmetry | 15.70 | 0.001 |
| Single limb support asymmetry | 15.70 | 0.001 |
| Initial mid swing asymmetry | 15.25 | 0.002 |
| Lumbar sagittal range of motion | 13.85 | 0.003 |
| Initial mid swing time | 13.77 | 0.003 |
| Trunk sagittal maximum angle | 13.76 | 0.003 |
| Foot phase difference var | 13.61 | 0.004 |
| Trunk sagittal average angle | 13.56 | 0.0036 |
| Terminal double support | 13.48 | 0.0037 |
| Trunk sagittal minimum angle | 13.19 | 0.0042 |
